# Supplementary material for: Dynamic transcriptomic profiles of zebrafish gills in response to zinc supplementation
Source: BMC Genomics. 2010 Oct 11;11:553. doi: 10.1186/1471-2164-11-553 (PMC3091702; doi:10.1186/1471-2164-11-553)
Supplement: Additional file 2 — Interactive Direct Interaction Network representing the molecular interactions between zinc, copper, iron, calcium and proteins encoded by transcripts changed by zinc supplementation. Mini web-site containing index.html and hyperlinked pages in subdirectory describing a Direct Interaction Network automatically generated based on curated interactions contained within the proprietary PathwayArchitect database. Ovals represent proteins and the circles symbolize metal ions. Objects are coloured by their abundance in zebrafish at the time-point they were significantly different from the control is a scale from -4 fold (dark green) to +4 fold (dark red). Where significant differences were found at more than one time-point, the colour overlay shows expression at the first instance. Dark blue squares denote 'binding', and light blue squares 'expression'; green squares stand for 'regulation', green diamonds for 'metabolism', and green circles for 'promoter binding'. Arrow heads indicate directionality of the interaction where annotated. All nodes and edges can be further interrogated by selecting the relative area of the image. [file 1471-2164-11-553-S2.zip › PathwayArchitect Zn xs DIN/754135.html]

# REGULATION:

|  |  |
| --- | --- |
| Type | REGULATION |
| Effect | Positive |


---

|  |  |
| --- | --- |
| Score | 0 |


---

|  |  |
| --- | --- |
| Reference Count | 7 |


---

|  |  |
| --- | --- |
| Mechanism | Unknown |


---

|  |  |
| --- | --- |
| Reference:0 || Sentence | "The effects of iron chelators, metabolic inhibitors and the cellular activators, lipopolysaccharide (LPS) and tumour necrosis factor-alpha (TNF-alpha), on Tf and Fe uptake were investigated." |
| PMID | 10865941 |
| Year | 2000 |
| Species | Mouse |
| Journal | Endothelium |
| RefScore | 0 |
| Source | PArchNLP |
  |
|  |  |


---

|  |  |
| --- | --- |
 Reference:1 || Sentence | "Atomic analysis of iron and gallium content of Sephadex G-150 fractions of treatment sera indicate that about an equimolar amount of gallium and iron are associated with transferrin." |
| PMID | 1337663 |
| Year | 1992 |
| Species | Human |
|  | Mouse |
| Journal | Am J Hematol |
| RefScore | 2 |
| Source | PArchNLP |
  ||  |  |


---

|  |  |
| --- | --- |
 Reference:2 || Sentence | "Early endosomal acidification appears to be normal in the mutant based on its accumulation of iron from transferrin and its sensitivity to diphtheria toxin A chain-transferrin conjugate." |
| PMID | 1645740 |
| Year | 1991 |
| Species | Mouse |
| Journal | J Cell Physiol |
| RefScore | 2 |
| Source | PArchNLP |
  ||  |  |


---

|  |  |
| --- | --- |
 Reference:3 || Sentence | "These mice provide a unique opportunity to examine the developmental pattern and response of Tf to iron-deficient diets, and furthermore, to address the controversial role of Tf in Mn transport." |
| PMID | 9850571 |
| Year | 1998 |
| Species | Mouse |
| Journal | Biometals |
| RefScore | 1 |
| Source | PArchNLP |
  ||  |  |


---

|  |  |
| --- | --- |
 Reference:4 || Sentence | RESULTS: Iron infusion increased total (Delta 601 microg/100 mL, CI 507, 696) and non-transferrin-bound iron (Delta 237.2 micromol/L, CI 173.6, 300.8) approximately 10-fold, as well as redox-active iron nearly five-fold (Delta 0.76 micromol/L, CI 0.54, 0.98). |
| Year | 2005 |
| PMID | 16316358 |
| Species | Human |
| Journal | Kidney Int |
| RefScore | 0 |
| Source | PArchNLP |
  ||  |  |


---

|  |  |
| --- | --- |
 Reference:5 || Sentence | Low-dose continuous iron therapy leads to a positive iron balance and decreased serum transferrin levels in chronic haemodialysis patients. |
| Year | 2004 |
| PMID | 15004263 |
| Species | Human |
| Journal | Nephrol Dial Transplant |
| RefScore | 2 |
| Source | PArchNLP |
  ||  |  |


---

|  |  |
| --- | --- |
 Reference:6 || Sentence | Higher iron stores, reflected by an elevated ferritin concentration and elevated transferrin saturation, can affect glucose intolerance during pregnancy. |
| PMID | 16079117 |
| Year | 2005 |
| Species | Human |
| Journal | Haematologica |
| RefScore | 2 |
| Source | PArchNLP |
  |


---

|  |  |
| --- | --- |
